# The Complete Chloroplast Genome of Chinese Bayberry (*Morella rubra*, Myricaceae): Implications for Understanding the Evolution of Fagales

**DOI:** 10.3389/fpls.2017.00968

**Published:** 2017-06-30

**Authors:** Lu-Xian Liu, Rui Li, James R. P. Worth, Xian Li, Pan Li, Kenneth M. Cameron, Cheng-Xin Fu

**Affiliations:** ^1^Laboratory of Plant Germplasm and Genetic Engineering, College of Life Sciences, Henan UniversityKaifeng, China; ^2^Key Laboratory of Conservation Biology for Endangered Wildlife of the Ministry of Education, College of Life Sciences, Zhejiang UniversityHangzhou, China; ^3^Food Inspection and Testing Institute of Henan ProvinceZhengzhou, China; ^4^Department of Forest Molecular Genetics and Biotechnology, Forestry and Forest Products Research InstituteIbaraki, Japan; ^5^Zhejiang Provincial Key Laboratory of Horticultural Plant Integrative Biology, Zhejiang UniversityHangzhou, China; ^6^Department of Botany, University of Wisconsin, MadisonWI, United States

**Keywords:** Fagales, *Morella rubra*, chloroplast genome, genomic structure, phylogenomics

## Abstract

*Morella rubra* (Myricaceae), also known as Chinese bayberry, is an economically important, subtropical, evergreen fruit tree. The phylogenetic placement of Myricaceae within Fagales and the origin of Chinese bayberry’s domestication are still unresolved. In this study, we report the chloroplast (cp) genome of *M. rubra* and take advantage of several previously reported chloroplast genomes from related taxa to examine patterns of evolution in Fagales. The cp genomes of three *M. rubra* individuals were 159,478, 159,568, and 159.586 bp in length, respectively, comprising a pair of inverted repeat (IR) regions (26,014–26,069 bp) separated by a large single-copy (LSC) region (88,683–88,809 bp) and a small single-copy (SSC) region (18,676–18,767 bp). Each cp genome encodes the same 111 unique genes, consisting of 77 different protein-coding genes, 30 transfer RNA genes and four ribosomal RNA genes, with 18 duplicated in the IRs. Comparative analysis of chloroplast genomes from four representative Fagales families revealed the loss of *inf*A and the pseudogenization of *ycf*15 in all analyzed species, and *rpl*22 has been pseudogenized in *M. rubra* and *Castanea mollissima*, but not in *Juglans regia* or *Ostrya rehderiana*. The genome size variations are detected mainly due to the length of intergenic spacers rather than gene loss, gene pseudogenization, IR expansion or contraction. The phylogenetic relationships yielded by the complete genome sequences strongly support the placement of Myricaceae as sister to Juglandaceae. Furthermore, seven cpDNA markers (*trn*H-*psb*A, *psb*A-*trn*K, *rps*2-*rpo*C2, *ycf*4-*cem*A, *pet*D-*rpo*A, *ndh*E-*ndh*G, and *ndh*A intron) with relatively high levels of variation and variable cpSSR loci were identified within *M. rubra*, which will be useful in future research characterizing the population genetics of *M. rubra* and investigating the origin of domesticated Chinese bayberry.

## Introduction

Chloroplasts (cp) are essential organelles in plant cells for photosynthesis and perform other functions comprising synthesizing starch, fatty acids, pigments and amino acids (Neuhaus and Emes, 2000). Typically, the sizes of chloroplast genomes and their gene arrangement in angiosperms are highly conserved and usually have a circular structure ranging from 120 to 160 kb, with two copies of inverted repeats (IR) region separated by a large single-copy (LSC) region and a small single-copy (SSC) region (Palmer, 1991; Raubeson and Jansen, 2005). Chloroplast genomes generally contain 110–130 distinct genes and these genes exhibit a highly conserved gene order with a majority of which (∼79) encoding proteins that are mostly involved in photosynthesis, whereas the rest of the genes encode approximately 30 transfer RNA (tRNA)s and four ribosomal RNA (rRNA)s (Jansen et al., 2005).

Compared with nuclear and mitochondrial genomes, chloroplast genomes are largely conserved in term of gene content, organization and structure (Raubeson and Jansen, 2005), and the nucleotide substitution rate of chloroplast genes is higher than that of mitochondrial genes, but lower than that of nuclear genes (Wolfe et al., 1987; Drouin et al., 2008). However, evolutionary events such as mutations, duplications, losses and rearrangements of genes have been reported in a number of studies (Lee et al., 2007; Dong et al., 2013; Choi et al., 2016). Due to its relatively small size, simple structure and conserved gene content, the chloroplast genome has been used as ideal research model for evolutionary and comparative genomic studies (Dong et al., 2013). In recent years, comparative studies of chloroplast genomes have been applied to a number of focal species (Young et al., 2011), genera (Greiner et al., 2008a,b), or plant families (Daniell et al., 2006). At higher taxonomic levels, comparative analyses of chloroplast genomes are useful for phylogenetic studies (Moore et al., 2007; Moore et al., 2010), as well as for understanding the genome evolution relating genome size variations, gene and intron losses and nucleotide substitutions. Moreover, chloroplasts have their own independent genome encoding an array of specific proteins, and the nature of non-recombinant and uniparental inheritance makes it a primarily useful tool in genomics and evolutionary research (Cho et al., 2015). Single nucleotide polymorphsims (SNPs) and indels, resulting from translocations, inversions, copy number variation of tandem repeats and rearrangements, are suitable for applying to phylogeny reconstruction (De Las Rivas et al., 2002), DNA barcoding (Hollingsworth et al., 2011), as well as investigating the geographic origin of some important domesticated crops (Arroyo-Garcia et al., 2006; Londo et al., 2006; Delplancke et al., 2013).

In this study, we analyzed the chloroplast genome of *Morella rubra* Lour. (Myricaceae), also known as Chinese bayberry, which is one of the most popular and valuable fruits in eastern China because of its appealing color, texture, delicious taste and nutritional value (Cheng et al., 2015). From the whole family Myricaceae, *M. rubra* is the only species to be domesticated as a fruit crop (Lu and Bornstein, 1999). Due to its long cultivation history (>2000 years) in China, as many as 305 accessions have been recorded, of which 268 have been named as cultivars (Zhang and Miao, 1999; Zhang et al., 2009). Wild populations of *M. rubra*, which are important germplasm resources for Chinese bayberry breeding, are distributed in the subtropical evergreen forests in China, Japan, South Korea and Philippines. Despite the economic importance of Chinese bayberry, its population genetics and domestication origin are still unclear. In fact, even the phylogenetic placement of *Morella* within Myricaceae, and the family within the order Fagales, remains ambiguous. This is one of the most economically and ecologically important flowering plant orders since it contains a number of domesticated nut and timber species, as well as dominant forest tree species (e.g., chestnut, walnut, hickory, oak, southern beech, birch).

Before 1990, Fagales was generally considered to contain only two families: Betulaceae and Fagaceae (Takhtajan, 1980; Cronquist, 1988). However, several large-scale phylogenetic analyses using DNA sequences (Chase et al., 1993; Soltis et al., 2000; Chen et al., 2016) and cpDNA restriction sites (Manos et al., 1993) have provided evidence for the monophyly of an expanded Fagales, which now comprises seven families: Nothofagaceae, Fagaceae, Myricaceae, Juglandaceae (including Rhoipteleaceae), Casuarinaceae, Ticodendraceae, and Betulaceae (APG III, 2009; APG IV, 2016). Most of the relationships within Fagales are well resolved, but the position of Myricaceae still remains uncertain. For example, some studies placed Myricaceae as sister to (Casuarinaceae + (Ticodendraceae + Betulaceae)) (Manos and Steele, 1997, *mat*K/*mat*K + *rbc*L; Cook and Crisp, 2005; Sauquet et al., 2012; Xiang et al., 2014; Sun et al., 2016), whereas others supported a sister relationship between Myricaceae and Juglandaceae (Li et al., 2004; Soltis et al., 2007; Larson-Johnson, 2016). Still others found that Myricaceae is sister to all Fagales except Nothofagaceae and Fagaceae (Manos and Steele, 1997, *rbc*L; Li et al., 2002). Thus, previous studies appear to have been based on insufficient information and thus could not fully resolve the phylogenetic position of Myricaceae.

Here, three individuals of *M. rubra* (Myricaceae) were selected for complete chloroplast genome sequencing. By comparing these three chloroplast genomes to each other and to previously published chloroplast genomes from other taxa in Fagales, we aim to: (1) characterize and compare the cp genomes among select representatives of Fagales in order to gain insights into evolutionary patterns within the order; (2) resolve the phylogenetic position of Myricaceae; (3) screen and identify appropriate markers of the *M. rubra* genome for future studies on population genetics and domestication origin.

## Materials and Methods

### DNA Sequencing and Genome Assembly

Total genomic DNA was isolated from silica-dried leaves of three wild *M. rubra* plants collected in Guangdong (GZMZ), Fujian (FJZS), and Yunnan (YNML) using a modified CTAB method (Li et al., 2013). The high molecular weight DNA was sheared using a Covaris S220-DNA Sonicator (Covaris, INC., Woburn, MA, United States), yielding fragments of ≤800 bp in length. The quality of fragmentation was checked on an Agilent Bioanalyzer 2100 (Agilent Technologies). Short-insert (500 bp) paired-end libraries were generated by using Genomic DNA Sample Prep Kit (Illumina) according to the manufacturer’s protocol and then sequenced using an Illumina HiSeq 2500 (Beijing Genomics Institute, Shenzhen, China). Resulting sequence fragments were screened by quality in order to remove low-quality sequences (Phred score <30, 0.001 probability error), and all remaining high quality sequences were assembled into contigs using the CLC *de novo* assembler beta 4.06 (CLC Inc., Rarhus, Denmark) with parameters as follows: minimum contig length of 200, deletion and insertion costs of 3, mismatch cost of 2, bubble size of 98, length fraction, and similarity fraction of 0.9. We obtained the principal contigs representing the chloroplast genome from the total assembled contigs using a BLAST (NCBI BLAST v2.2.31) search with the cp genome sequence of *J. regia* (GenBank accession number: KT870116) as a reference sequence (Peng et al., 2015). The representative chloroplast sequence contigs were ordered and oriented according to the reference chloroplast genome, and the complete chloroplast sequence of *M. rubra* was constructed by connecting overlapping terminal sequences.

### Genome Annotation and Molecular Marker Identification

The cp genomes of *M. rubra* were annotated through the online program Dual Organellar Genome Annotator (DOGMA; Wyman et al., 2004). Initial annotation, putative starts, stops, and intron positions were determined according to comparisons with homologous genes of *J. regia* and *Castanea mollissima* (GenBank accession number: HQ336406) cp genomes using Geneious v9.0.5 software (Biomatters, Auckland, New Zealand). In addition, all of the identified tRNA genes were further verified by using the corresponding structures predicted by tRNAscan-SE version 1.21 (Schattner et al., 2005) with default settings. The cp genome map of *M. rubra* was constructed utilizing the OGDRAW program (Lohse et al., 2013).

The three completed chloroplast genome sequences of *M. rubra* were aligned using MAFFT (Katoh et al., 2002). In order to screen various polymorphic regions among individuals of *M. rubra* (i.e., below the species level), the average number of nucleotide differences (K) and total number of mutations (Eta) were determined to analyze nucleotide diversity (Pi) using DnaSP v5.0 (Librado and Rozas, 2009).

### Repeat Structure and Sequence Analysis

We used the online REPuter software to visualize and locate forward, palindrome, reverse and complement sequences with a minimum repeat size of 30 bp and a sequence identity greater than 90% (Kurtz and Schleiermacher, 1999).

Microsatellite (mono-, di-, tri-, tetra-, penta-, and hexanucleotide repeats) detection was performed using Msatcommander v0.8.2 (Faircloth, 2008). We applied a threshold nine, five, five, three, three, and three repeat units for mono-, di-, tri-, tetra-, penta-, and hexanucleotide SSRs, respectively.

### Comparative Chloroplast Genomic Analysis

We downloaded *Castanea mollissima, Juglans regia*, and *Ostrya rehderiana* (GenBank accession number: KT454094) chloroplast genome sequences from GenBank, in order to compare the overall similarities among different chloroplast genomes in Fagales. Pairwise alignments among four Fagales cp genomes were implemented in the mVISTA program with LAGAN mode (Frazer et al., 2004) using the annotation of *Cucumis sativus* (Cucurbitaceae, Cucurbitales; GenBank accession number: DQ865976) as the reference.

### Synonymous (*K*_S_) and Non-synonymous (*K*_A_) Substitution Rates Analysis

The DnaSP v5.0 (Librado and Rozas, 2009) software was employed to analyze the relative rates of sequence divergence in the four Fagales species and the reference sequence. In order to analyze synonymous (K_S_) and non-synonymous (K_A_) substitution rates, we extracted the same individual functional protein-coding exons and aligned separately using Geneious v9.0.5. Genes with the same functions were grouped and analyses were carried out on (1) datasets corresponding to those with the same functions, i.e., for *atp, pet, ndh, psa, psb, rpl, rpo*, and *rps*; (2) datasets corresponding to singular genes, i.e., for *cem*A, *mat*K, *ccs*A, *clp*P, *rbc*L, and *ycf*1; and (3) concatenated common protein-coding genes, except for pseudogenes or lost genes from any species.

### Phylogeny Inference

The complete chloroplast genome sequences of eight species from Fagales (10 accessions) were used for phylogenetic analysis, including representatives of five genera of Fagaceae, one genus of Betulaceae, one genus of Juglandaceae, and the three newly sequenced individuals of *M. rubra* used to represent Myricaceae (Supplementary Table S1). Two species from Cucurbitales (*Corynocarpus laevigata* and *Cucumis sativus*) were chosen as outgroup taxa to orient the Fagales tree. In order to investigate the utility of different regions, the phylogeny was inferred using two datasets: (1) the complete chloroplast genome sequences; and (2) a set of 69 protein-coding genes shared by the chloroplast genomes of the 12 accessions. All the gaps were excluded after alignment in both analyses.

All phylogenetic analyses were conducted using maximum-likelihood (ML) and Bayesian inference (BI) methods. ML analyses were implemented in RAxML-HPC v8.1.11 on the CIPRES cluster^1^ (Miller et al., 2010) using the best-fit nucleotide substitution model (GTR+I+G) determined from jModelTest v2.1.4 (Posada, 2008) for the cp genome dataset and a partitioned model for protein-coding regions. BI analyses were performed in MrBayes v3.2.3 (Ronquist and Huelsenbeck, 2003) using the same model selection criteria for both data sets. Two independent parallel runs of four Metropolis-coupled Monte Carlo Markov Chains (MCMCs) were run with trees sampling every 1000 generations for five million total generations.

## Results and Discussion

### Genome Content and Organization in *M. rubra*

We generated a total of 8.5 million paired-end (PE) reads (200 million nucleotides) for *M. rubra*-GZMZ, and then trimmed and assembled them using the CLC genome assembler pipeline (CLC Bio, Aarhus, Denmark). A total of 290,501 PE reads were concordantly mapped to the final assembly and the mapped cp contigs were selected to merge for constructing a complete *M. rubra*-GZMZ cp genome map using BLAST (NCBI BLAST v2.2.31). Four initial contigs (contigs 16, 39, 79, and 883 respectively) were selected to generate the *M. rubra*-GZMZ cp genome sequence with no gaps and no Ns. The cp genome sequence was registered into GenBank with the accession number KY476637.

**FIGURE 1:**
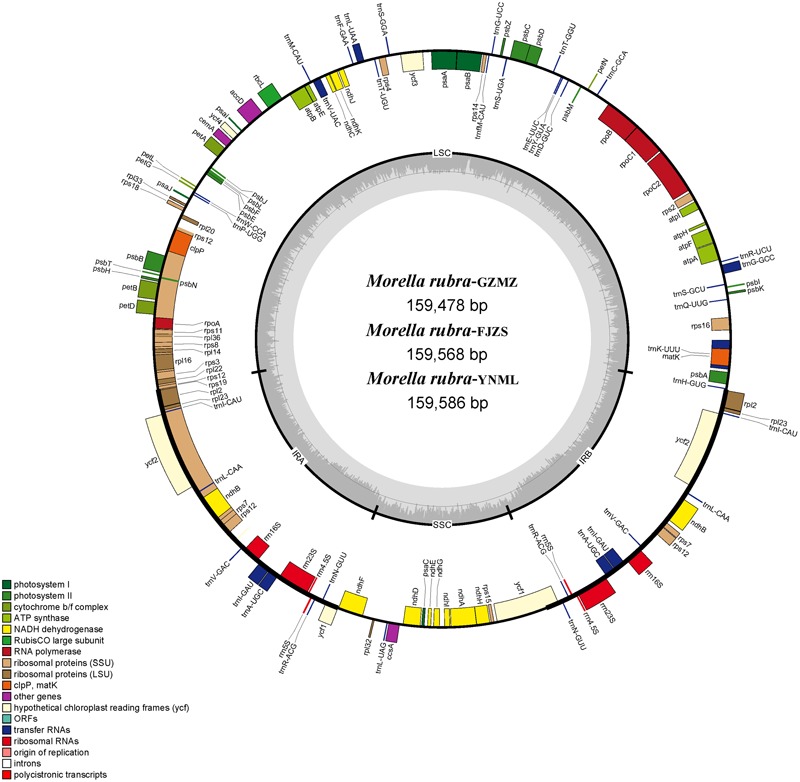
Chloroplast genome map of *Morella rubra* (Myricaceae). The genes inside and outside of the circle are transcribed in the counterclockwise and clockwise directions, respectively. Genes belonging to different functional groups are shown in different colors. The thick lines indicate the extent of the inverted repeats (IR_A_ and IR_B_) that separate the genomes into small single-copy (SSC) and large single-copy (LSC) regions.

The complete chloroplast genome of *M. rubra*-GZMZ is 159,478 bp in length and shares the common feature of comprising two copies of IR (26,014 bp each) that divide the genome into two single-copy regions (LSC 88,683 bp; SSC 18,767 bp; **Figure 1**). The overall GC content of the total length, LSC, SSC, and IR regions is 36.1, 33.8, 29.2, and 42.6%, respectively. Coding regions (91,795 bp), comprising protein-coding genes (79,949 bp), tRNA genes (2,798 bp) and rRNA genes (9,048 bp) account for 57.56% of the genome, whereas non-coding regions (67,683 bp), including intergenic spaces (49,558 bp) and introns (18,125 bp) account for the remaining 42.44% of the genome.

Within the chloroplast genome of *M. rubra* there are in total 111 genes, including 77 protein-coding genes, 30 tRNA genes, four rRNA genes and 18 duplicated genes (**Figure 1** and **Tables 1, 2**). Among the 111 unique genes, 15 contain one intron (six tRNA genes and nine protein-coding genes) and three (*rps*12, *clp*P, and *ycf*3) contain two introns. The 5′-end exon of the *rps*12 gene is located in the LSC region, and the intron and 3′-end exon of the gene are situated in the IR region. In addition to the GZMZ accession, we also sequenced the complete cp genomes of *M. rubra*-FJZS (GenBank accession number: KY476636) and *M. rubra*-YNML (GenBank accession number: KY476635). These are 159,568 and 159,586 bp in size, respectively, and the genome content and organization of them is nearly the same as the cp genome of *M. rubra*-GZMZ (**Figure 1** and **Table 1**).

**Table 1:** Comparative analysis of the chloroplast genomes among four families of Fagales, including three different accessions of *Morella rubra* (Myricaceae) sequenced for this study.

	*M. rubra*-GZMZ	*M. rubra*-FJZS	*M. rubra*-YNML	*Juglans regia*	*Castanea mollissima*	*Ostrya rehderiana*
Total cpDNA size	159,478	159,568	159,586	160,537	160,799	159,347
Length of large single copy (LSC) region	88,683	88,809	88,772	90,059	90,432	88,177
Length of inverted repeat (IR) region	26,014	26,015	26,069	26,033	25,686	26,131
Length of small single copy (SSC) region	18,767	18,706	18,676	18,412	18,995	18,908
Coding size	91,795	91,239	91,818	90,810	90,465	91,041
Intron size	20,647	20,667	20,705	20,712	19,957	20,640
Spacer size	47,036	47,662	47,063	49,015	50,377	47,666
Total GC content (%)	36.10	36.10	36.10	36.20	36.80	36.50
LSC	33.80	33.80	33.80	33.60	34.60	34.30
IR	42.60	42.60	42.60	42.60	42.80	42.50
SSC	29.20	29.20	29.20	29.80	30.80	29.80
Total number of genes	111	111	111	113	111	112
Protein encoding	77	77	77	80	77	78
tRNA	30	30	30	30	30	30
rRNA	4	4	4	4	4	4
Number of genes duplicated in IR	18	18	18	17	16	17

**Table 2:** List of genes present in the *M. rubra* chloroplast genome.

Category	Gene group	Gene name			
Self-replication	Ribosomal RNA genes	*rrn*4.5^a^	*rrn*5^a^	*rrn*16^a^	*rrn*23^a^
	Transfer RNA genes	*trnA*-UGC^a,b^ *trnF-*GAA *trnH-*GUG *trnL-*CAA^a^ *trnN-*GUU^a^ *trnR-*UCU *trnT-*GGU *trnW-*CCA	*trnC-*GCA *trnfM*-CAU *trnI-*CAU^a^ *trnL-*UAA^b^ *trnP-*UGG *trnS-*GCU *trnT-*UGU *trnY-*GUA	*trnD-*GUC *trnG-*GCC *trnI-*GAU^a,b^ *trnL-*UAG *trnQ-*UUG *trnS-*GGA *trnV-*GAC^a^	*trnE-*UUC *trnG-*UCC^b^ *trnK-*UUU^b^ *trnM-*CAU *trnR-*ACG^a^ *trnS-*UGA *trnV-*UAC^b^
	Small subunit of ribosome	*rps*2 *rps8* *rps*15	*rps*3 *rps*11 *rps*16^b^	*rps*4 *rps*12^a,c,d^ *rps*18	*rps*7^a^ *rps*14 *rps*19
	Large subunit of ribosome	*rpl*2^a,b^ *rpl*23^a^	*rpl*14 *rpl*32	*rpl*16^b^ *rpl*33	*rpl*20 *rpl*36
	DNA-dependent RNA polymerase	*rpo*A	*rpo*B	*rpo*C1^b^	*rpo*C2
Photosynthesis	Subunits of photosystem I	*psa*A *psa*J	*psa*B *ycf*3^c^	*psa*C *ycf*4	*psa*I
	Subunits of photosystem II	*psb*A *psb*E *psb*J *psb*N	*psb*B *psb*F *psbK* *psb*T	*psb*C *psb*H *psb*L *psb*Z	*psb*D *psb*I *psb*M
	Subunits of cytochrome	*pet*A *pet*L	*pet*B^b^ *pet*N	*pet*D^b^	*pet*G
	Subunits of ATP synthase	*atp*A *atp*H	*atp*B *atp*I	*atp*E	*atp*F^b^
	Large subunit of Rubisco	*rbc*L			
	Subunits of NADH Dehydrogenase	*ndh*A^b^ *ndh*E *ndh*I	*ndh*B^a,b^ *ndh*F *ndh*J	*ndh*C *ndh*G *ndh*K	*ndh*D *ndh*H
Other genes	Maturase	*mat*K			
	Envelope membrane protein	*cem*A			
	Subunit of acetyl-CoA	*acc*D			
	C-type cytochrome synthesis gene	*ccs*A			
	Protease	*clp*P^c^			
	Proteins of unknown function	*ycf*1^a^	*ycf*2^a^		
Pseudogenes		*ycf*15	*rpl*22		

*^a^Two gene copies in IRs; ^b^gene containing a single intron; ^c^gene containing two introns; ^d^gene divided into two independent transcription units.*

### Genome Organization of Fagales

**FIGURE 2:**
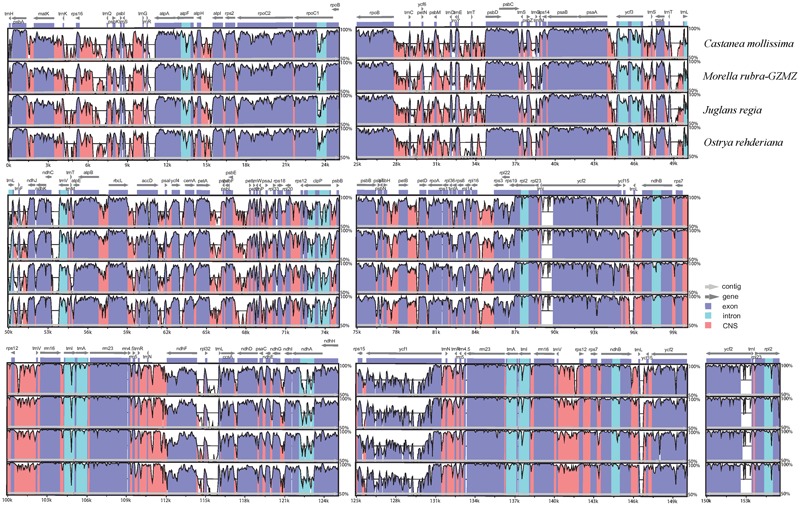
Identity plot comparing the chloroplast genomes of four Fagales families using *Cucumis sativus* as the reference sequence. The vertical scale indicates the percentage of identity, ranging from 50 to 100%. The horizontal axis indicates the coordinates within the chloroplast genome. Genome regions are color codes as protein-coding, rRNA, tRNA, intron, and conserved non-coding sequences (CNS).

The chloroplast genome organization is rather conserved within Fagales (**Figure 2**). We did not detect either translocations or inversions among any of the compared genomes. The IR region in these species is more conserved than the LSC and SSC regions, consistent with other angiosperms (Dong et al., 2013;Lu R. et al., 2016). Variations were detected with the following factors: genome size, gene losses, the pseudogenization of protein-coding genes, and IR expansion and contraction.

#### Genome Size

Among the representative Fagales species, *O.rehderiana* exhibits the smallest genome size after comparing with the other three chloroplast genomes. The genome of *Castanea mollissima* (160,799 bp) is approximately 1.45 kb larger than that of *O. rehderiana*, 1.32 kb larger than that of *M. rubra*, and 0.26 kb larger than that of *J. regia*, as well as it is 5.28 kb larger than that of *Cucumis sativus*, an outgroup species. The detected sequence length difference is predominantly attributable to the variation in the length of the non-coding regions, especially in terms of intergenic spacer size (**Table 1**). The *M. rubra*-GZMZ genome exhibits the smallest non-coding region among the six analyzed chloroplast genomes.

#### Gene Loss

A single gene, *inf*A, has been lost from all the four analyzed chloroplast genomes. After comparisons with the chloroplast genomes of other Fagales species, this gene also appears to be missing in *Castanea pumila* (GenBank accession number: KM360048) and *Trigonobalanus doichangensis* (GenBank accession number: NC023959), although it is present in *Quercus edithiae* (GenBank accession number: KU382355), *Q. rubra* (GenBank accession number: NC020152), *Castanopsis echinocarpa* (GenBank accession number: NC023801), *Lithocarpus balansae* (GenBank accession number: NC026577), *Q. aliena* (Lu S. et al., 2016), *Q. spinosa* (GenBank accession number: NC026907), *Q. aquifolioides* (GenBank accession number: NC026913), and *Q. baronii* (GenBank accession number: NC029490). *Inf*A gene was thought to have functions as a translation initiation factor, which assists in the assembly of the translation initiation complex (Wicke et al., 2011). This gene is also possibly transferred to the nucleus and loss of which appears to have independently occurred multiple times during the evolution of land plants (Millen et al., 2001). Dong et al. (2013) reported the two genes including *inf*A and *rpl*32 had been lost from the chloroplast genome of *Paeonia obovata*. Therefore, the loss of *inf*A does not represent a unique phenomenon in some species of Fagales.

#### Gene Pseudogenization

*ycf*15 has been pseudogenized in all four representatives of Fagales, and *rpl*22 has been pseudogenized in *M. rubra* and *Castanea mollissima* but not in *J. regia* and *O. rehderiana*. The *ycf*15 gene, which has been paid great attention to its function by previous workers (Raubeson et al., 2007; Shi et al., 2013), is located immediately downstream of the *ycf*2 gene (Dong et al., 2013). Some studies have shown that the *ycf*15 gene is potentially functional (Shinozaki et al., 1986), but the validity of *ycf*15 as a protein-coding gene in angiosperms has long been questioned (Tangphatsornruang et al., 2011). The *ycf*15 presents a pseudogene in all the sequenced chloroplast genome of Fagales except *Q. rubra*. In Fagales, *rpl*22 appears as a pseudogene in Myricaceae and Fagaceae because there remain some internal stop codons within the coding region, and not to be pseudogenized in Juglandaceae and Betulaceae. Jansen et al. (2011) reported that *rpl*22 has been transferred to the nucleus in Fagaceae, whether the *rpl*22 gene has been transferred to the nucleus in Myricaceae remains to be investigated.

#### IR Expansion and Contraction

**FIGURE 3:**
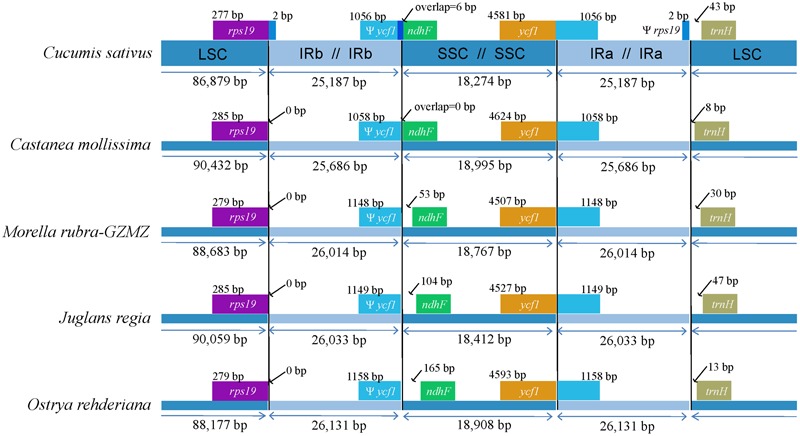
Comparison of junction positions between the single copy and IR regions among four Fagales genomes and *Cucumis sativus*.

The expansions and contractions of the IR regions and the single-copy (SC) boundary regions often results in genome size variations among various plant lineages (Wang et al., 2008), and may reflect phylogenetic history. For this reason, we paid careful attention to the exact IR/SC border positions and their adjacent genes among the four Fagales species chloroplast genomes that we studied in detail (**Figure 3**). The *ycf*1 gene spanned the SSC/IR_A_ region and the pseudogene fragment of ψ*ycf*1 varies from 1058 to 1158 bp. The *ndh*F gene is separated from ψ*ycf*1 by spacers except in *Castanea mollissima* which does not contain a spacer (53 bp in *M. rubra*, 104 bp in *J. regia* and 165 bp in *O. rehderiana*) but shares some nucleotides (6 bp) with the *ycf*1 pseudogene in our outgroup taxon, *Cucumis sativus*. The *trn*H-GUG gene is generally located downstream of the IR_A_/LSC border, and this gene is separated from the IR_B_/LSC border by a spacers varies from 8 to 47 bp. However, the *rps*19 gene does not extend to the IR region among the sampled representatives of Fagales. Thus, the *rps*19 pseudogene is not observed in Fagales. Although there are expansions and/or contractions of the IR regions detected among the sampled representatives of Fagales, they contribute little to the overall size variations in the chloroplast genomes of these plants.

### Repeat Sequence Analysis and Molecular Marker Identification

**FIGURE 4:**
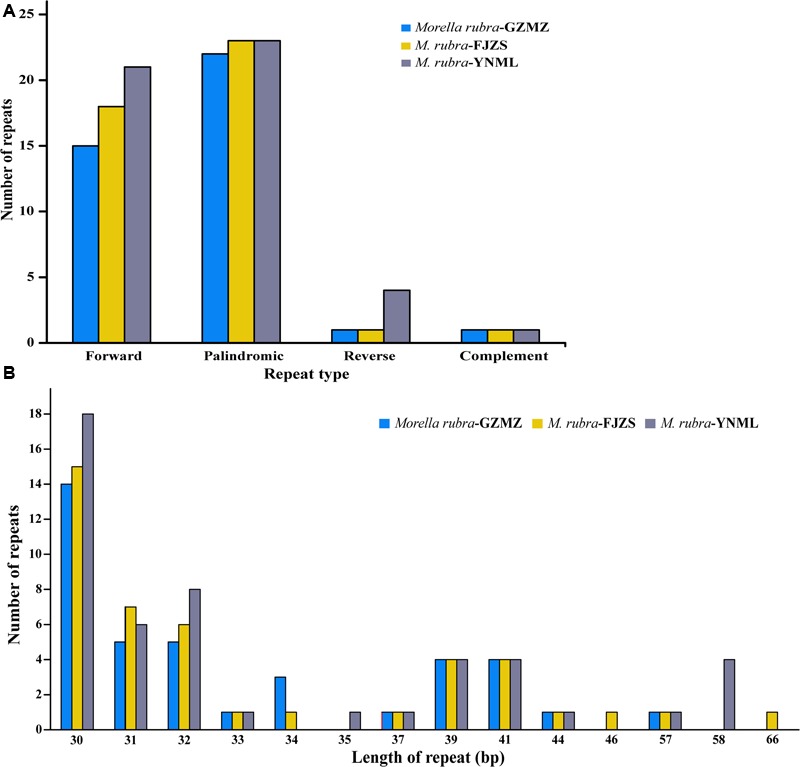
Analysis of repeated sequences in the three *M. rubra* chloroplast genomes. **(A)** Frequency of repeat types. **(B)** Frequency of repeats by length.

Repeat motifs are thought to play an important role in phylogenetic studies and are very useful in the analysis of genome rearrangement (Cavalier-Smith, 2002; Nie et al., 2012). In the chloroplast genome of *M. rubra*-GZMZ, 39 pairs of repeats (30 bp or longer) containing 22 palindromic repeats, 15 forward repeats, one complement repeat and one reverse repeat were detected using the program REPuter (Kurtz and Schleiermacher, 1999) (**Figure 4A**). Among these repeats, 33 are 30–40 bp long, four repeats are 41 bp long, one repeat is 44 bp long and one repeat is 57 bp long (**Figure 4B**). Most of these repeats (53.8%) are distributed in non-coding regions (**Table 3**), whereas some are found in genes such as *ycf*1, *ycf*2, *ycf*3, *psa*B, and *pas*A. Further information about the repeat motifs of *M. rubra*-FJZS and *M. rubra*-YNML can be found in Supplementary Tables S2, S3.

**Table 3:** Repeated sequences in the *M. rubra*-GZMZ chloroplast genome.

Repeat no.	Repeat size (bp)	Repeat start 1	Repeat start 2	Type	Location of repeat 1	Location of repeat 2
1	30	136188	136219	F	*rrn*5S/*rrn*4.5S^∗^	*rrn*5S/*rrn*4.5S^∗^
2	30	133974	133974	P	*ycf*1	*ycf*1
3	30	114157	114157	P	*ycf*1	*ycf*1
4	30	114157	133974	F	*ycf*1	*ycf*1
5	30	111943	136219	P	*rrn*4.5S/*rrn*5S^∗^	*rrn*5S/*rrn*4.5S^∗^
6	30	111912	111943	F	*rrn*4.5S/*rrn*5S^∗^	*rrn*4.5S/*rrn*5S^∗^
7	30	111912	136188	P	*rrn*4.5S/*rrn*5S^∗^	*rrn*5S/*rrn*4.5S^∗^
8	30	47244	102983	F	*ycf*3	*rps*12/*trn*V-GAC^∗^
9	30	47244	145148	P	*ycf*3	*trn*V-GAC/*rps*12^∗^
10	30	42178	44402	F	*psa*B	*psa*A
11	30	39386	39402	F	*psb*Z/*trn*G-GCC^∗^	*psb*Z/*trn*G-GCC^∗^
12	30	38558	49028	P	*trn*S-UGA	*trn*S-GGA
13	30	34469	34469	P	*trn*T-GGU/*psb*D^∗^	*trn*T-GGU/*psb*D^∗^
14	30	9267	49028	P	*trn*S-GCU	*trn*S-GGA
15	31	126455	126455	P	*ndh*A (intron)	*ndh*A (intron)
16	31	117134	117134	P	*ndh*F/*rpl*32^∗^	*ndh*F/*rpl*32^∗^
17	31	34994	118170	C	*trn*T-GGU/*psb*D^∗^	*rpl*32/*trn*L-UAG^∗^
18	31	34994	118161	R	*trn*T-GGU/*psb*D^∗^	*rpl*32/*trn*L-UAG^∗^
19	31	9263	38554	F	*trn*S-GCU	*psb*C/*trn*S-UGA^∗^
20	32	154596	154617	F	*ycf*2	*ycf*2
21	32	132804	132804	P	*ycf*1	*ycf*1
22	32	93533	154617	P	*ycf*2	*ycf*2
23	32	93512	93533	F	*ycf*2	*ycf*2
24	32	93512	154596	P	*ycf*2	*ycf*2
25	33	125664	125664	P	*ndh*A (intron)	*ndh*A (intron)
26	34	125872	125878	P	*ndh*A (intron)	*ndh*A (intron)
27	34	124227	124227	P	*ndh*G/*ndh*I^∗^	*ndh*G/*ndh*I^∗^
28	34	16216	16228	F	*atp*H/*atp*I^∗^	*atp*H/*atp*I^∗^
29	37	47232	125426	F	*ycf*3	*ndh*A (intron)
30	39	125424	145153	P	*ndh*A (intron)	*trn*V-GAC/*rps*12^∗^
31	39	102969	125424	F	*rps*12/*trn*V-GAC^∗^	*ndh*A (intron)
32	39	47232	102971	F	*ycf*3	*rps*12/*trn*V-GAC^∗^
33	39	47232	145151	P	*ycf*3	*trn*V-GAC/*rps*12^∗^
34	41	152170	152188	F	*ycf*2	*ycf*2
35	41	95950	152188	P	*ycf*2	*ycf*2
36	41	95932	95950	F	*ycf*2	*ycf*2
37	41	95932	152170	P	*ycf*2	*ycf*2
38	44	78648	78648	P	*psb*T/*psb*N^∗^	*psb*T/*psb*N^∗^
39	57	6935	6935	P	*rps*16/*trn*Q-UUG^∗^	*rps*16/*trn*Q-UUG^∗^

*Type are F (Forward), P (palindromic), C (complement), and R (reverse) repeats.*

*^∗^intergenic space.*

**FIGURE 5:**
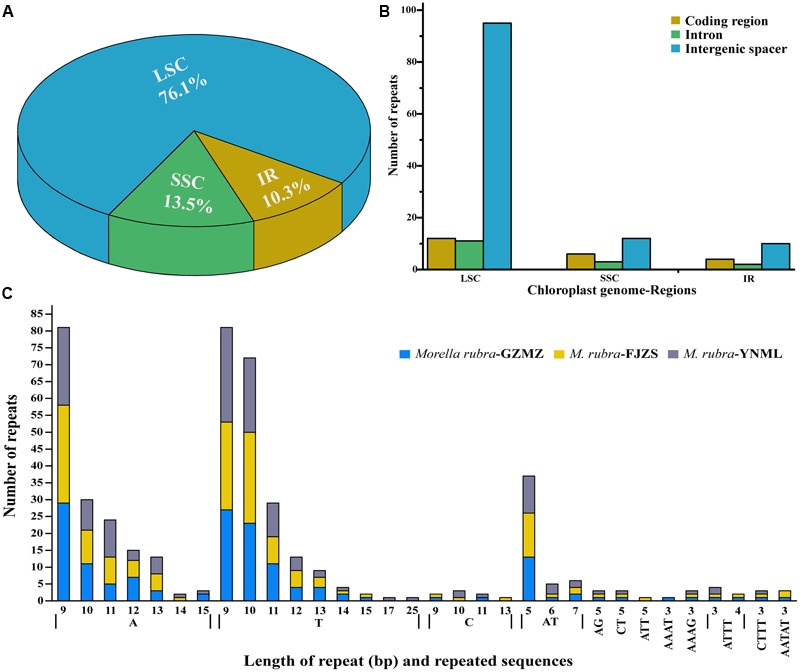
The distribution, type, and presence of simple sequence repeats (SSRs) in the cp genome of *M. rubra*. **(A)** Presence of SSRs in the LSC, SSC, and IR regions (*M. rubra*-GZMZ). **(B)** Presence of SSRs in the protein-coding regions, intergenic spacers and introns of LSC, SSC, and IR regions (*M. rubra*-GZMZ). **(C)** Presence of polymers in the cp genome of *M. rubra*.

Simple sequence repeats (SSR), also known as microsatellites, are widely distributed over the genome (Chen et al., 2015) and have a high degree of polymorphism (Weber, 1990). As a result, SSRs are widely used as a molecular marker for breeding (Rafalski and Tingey, 1993), population genetics (Perdereau et al., 2014), genetic linkage map construction, and gene mapping (Pugh et al., 2004). In the current study, the distribution, type and presence of microsatellites were studied among the cp genomes of three *M. rubra* accessions. We did this, in part, because we are interested in developing markers that may be useful in future studies that will address intraspecific variation among natural populations and cultivars of *M. rubra* across East Asia. A total of 155 perfect microsatellites were identified in the *M. rubra*-GZMZ cp genome. Among them, 118 were located in the LSC regions, whereas 16 and 21 were found in the IR and SSC regions, respectively (**Figure 5A**). In addition, 22 SSRs were found in the protein-coding regions, 16 were in the introns and 117 were in intergenic spacers of the *M. rubra*-GZMZ cp genome (**Figure 5B**). The distribution and type of microsatellites of *M. rubra*-FJZS and *M. rubra*-YNML is shown in **Supplementary Figure S1**. Among these SSRs, 131 are mononucleotides, 18 are dinucleotides, five are tetranucleotides, and one is a pentanucleotide (**Figure 5C**). Trinucleotide SSRs are not found in *M. rubra*-GZMZ or *M. rubra*-YNML but were detected in *M. rubra*-FJZS. A majority of the mononucleotides (98.47%) are composed of A/T and most of the dinucleotides (88.89%) are composed of AT/TA (**Figure 5C**). These results are consistent with the contention that cp SSRs are generally composed of short polyA or polyT repeats (Kuang et al., 2011; Chen et al., 2015). The higher A/T content in cp SSRs also contributes to a bias in base composition, resulting in A/T enrichment (63.9%) in the *M. rubra*-GZMZ cp genome.

The coding genes, non-coding regions and intron regions were compared among the three individuals of *M. rubra* divergence hotspots. We generated 90 loci (28 coding genes, 52 intergenic spacers, and 10 intron regions) with more than 200 bp in length from three *M. rubra* individuals and the nucleotide variability (*P*i) values calculated with the DnaSP v5.0 software.

**FIGURE 6:**
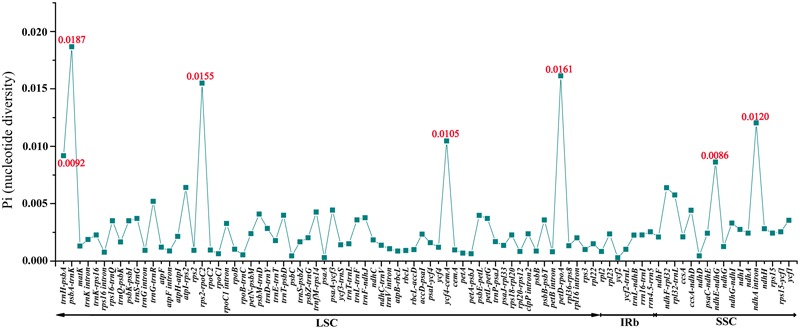
Comparative analysis of the nucleotide variability (*P*i) values among three *M. rubra* individuals.

Among the values received from the three individuals of *M. rubra* (*M. rubra*-GZMZ, *M. rubra*-FJZS, and *M. rubra*-YNML) ranged from 0.00029 (*ycf*2 gene) to 0.01867 (*psb*A*-trn*K region) (**Figure 6**). The IR region is much more conserved than the LSC and SSC regions, and the lower sequence divergence observed in the IRs compared to the SSC or LSC regions for *Morella* species and other angiosperms is likely due to copy correction between IR sequences by gene conversion (Khakhlova and Bock, 2006; Lu R. et al., 2016). Seven of these variable loci, including *trn*H-*psb*A, *psb*A-*trn*K, *rps*2-*rpo*C2, *ycf4*-*cem*A, *pet*D-*rpo*A, *ndh*E-*ndh*G, and *ndh*A intron, showed high levels of variation. Five of them (*trn*H-*psb*A, *psb*A-*trn*K, *rps*2-*rpo*C2, *ycf*4-*cem*A, and *pet*D-*rpo*A) are located in the LSC, whereas two (*ndh*E-*ndh*G and *ndh*A intron) are in the SSC region (**Figure 6**).

All seven of these variable loci (*trn*H-*psb*A, *psb*A*-trn*K, *rps*2-*rpo*C2, *ycf*4-*cem*A, *pet*D-*rpo*A, *ndh*E-*ndh*G, and *ndh*A intron) show great potential as highly informative phylogenetic markers in *M. rubra*. The results presented here will be helpful to the study on the domestication origin of Chinese bayberry in the future.

### Synonymous (*K*_S_) and Non-synonymous (*K*_A_) Substitution Rate Analysis

Nonsynonymous (*K*_A_) and synonymous (*K*_S_) substitutions and their ratio (*K*_A_/*K*_S_) are important to indicate the rates of evolution and natural selection (Yang and Nielsen, 2000). Synonymous nucleotide substitutions have occurred more frequently than nonsynonymous substitutions, and the *K*_A_/*K*_S_ value is usually less than one in most protein-coding regions (Makalowski and Boguski, 1998). In this study, these parameters were compared among the protein-coding chloroplast genes of the four-representative species of Fagales to investigate genome evolution, with the cp genome of *Cucumis sativus* as a reference (**Table 4**). The *K*_A_ values of the four-representative species ranged from 0.0879 to 0.0962, as well as the *K*_S_ values ranged from 0.01489 to 0.01605. Both the *K*_A_ and *K*_S_ values consistently indicated that *Castanea mollissima* has evolved a little rapidly than the other three species in Fagales. The *K*_A_/*K*_S_ values of these Fagales species are less than 1, providing the evidence of purifying selection on the chloroplast protein-coding genes of Fagales species.

**Table 4:** Substitution rates of 75 protein-coding genes in four Fagales chloroplast genomes.

Taxa	Nonsynonymous (K_A_)	Synonymous (*K*_S_)	*K*_A_/*K*_S_
*Morella rubra*	0.0901 ± 0.0196	0.1547 ± 0.0258	0.7561
*Juglans regia*	0.0889 ± 0.0205	0.1489 ± 0.0234	0.7442
*Castanea mollissima*	0.0962 ± 0.0217	0.1605 ± 0.0248	0.7859
*Ostrya rehderiana*	0.0879 ± 0.0188	0.1556 ± 0.0239	0.7248

*Cucumis sativus was used as an outgroup. Data are presented as the means ± standard error.*

**FIGURE 7:**
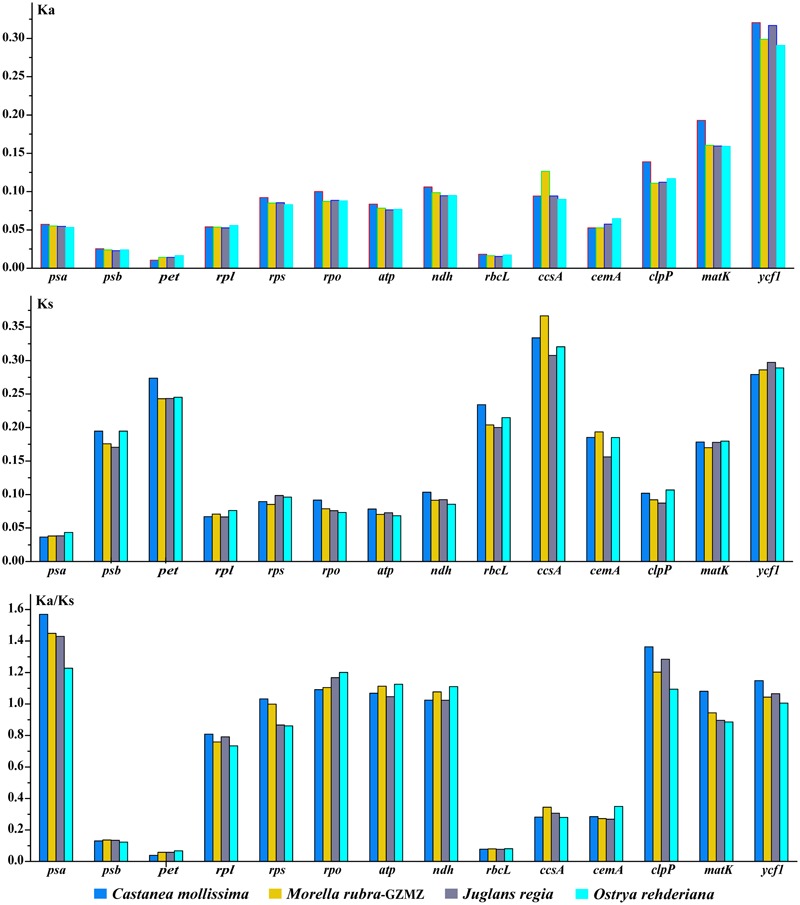
Non-synonymous substitution (*K*_A_), synonymous substitution (*K*_S_), and *K*_A_/*K*_S_ values for individual Fagales genes and groups of genes. The *rpl*22 is not included in the *rpl* group due to its pseudogenization in some species.

Variations in evolutionary rates can be related to the function of genes and genome structure (Chang et al., 2006; Jansen et al., 2007; Dong et al., 2013). In Fagales species, the four-sampled genome structure are quite conserved, without any remarkable restructuring being detected. Comparing with the outgroup *Cucumis sativus*, the *K*_A_ (*F* = 293.17, *P* < 0.001) and *K*_S_ (*F* = 245.86, *P* < 0.001) values shown differ significantly among gene groups classified according to gene functions (**Figure 7**). The *psb, pet*, and *rbc*L genes show the lowest *K*_A_ values, while the *ycf*1 gene exhibits the highest *K*_A_ values. Moreover, the *psa* gene shows the lowest *K*_S_ values, whereas *ccs*A gene exhibits the highest *K*_S_ values. According to the *K*_A_/*K*_S_ values, we found that the *psa, rpo, atp, clp*P, and *ycf*1 genes are under positive selection in Fagales.

### Phylogeny Inference

**FIGURE 8:**
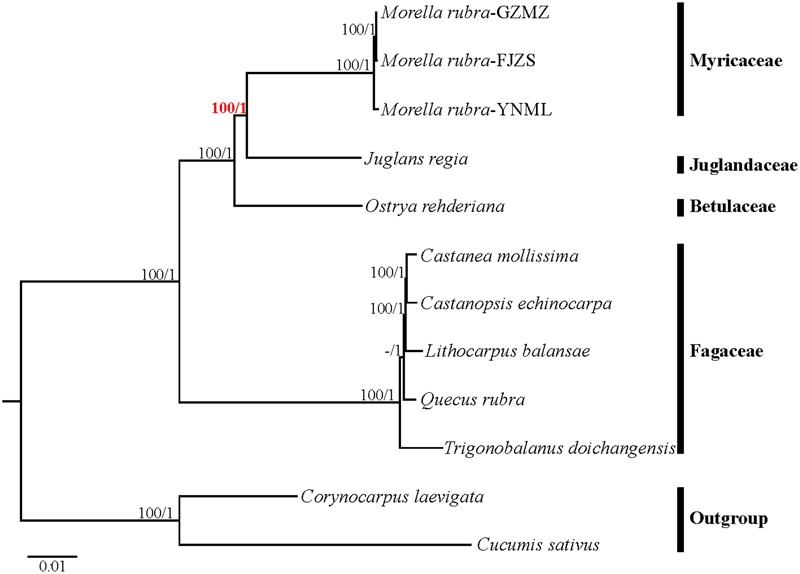
Phylogenetic tree reconstruction of Fagales using maximum likelihood (ML) based on whole chloroplast genome sequences. Relative branch lengths are indicated. Numbers above the lines represent ML bootstrap values / BI posterior probability. The hyphen indicates that a ML bootstrap <50%. A phylogenetic tree resulting from analysis of 69 protein-coding genes was fully congruent with this topology.

Relationships within Fagales are fairly well resolved in previously published studies, but the position of Myricaceae still remains somewhat uncertain (Manos and Steele, 1997; Cook and Crisp, 2005; Li et al., 2016). Most of these earlier studies have used sequences from only one or more chloroplast loci. In the present study, we explored two datasets: the complete chloroplast genome and a restricted matrix of 69 commonly shared protein-coding genes to perform phylogenetic analysis. For the analysis with the complete chloroplast genome data, the tree topologies from both the ML and the Bayesian analysis were found to be consistent with each other (**Figure 8**). All the analyzed families within Fagales have MLBS = 100%. Fagaceae are sister to the remaining Fagales (MLBS = 100%), followed by Betulaceae, which are subsequently sister to the remainder of the Fagales, with full support (MLBS = 100%). The remaining two families, Juglandaceae and Myricaceae, form one clade with BS = 100%, as well as the three Myricaceae individuals forming one clade with MLBS = 100%. The relationships among them are identical with the system of classification proposed by APG III (APG III, 2009).

Most phylogenomic studies have not used entire plastome sequences, but rather have used a subset of common protein coding genes (Jansen et al., 2007; Moore et al., 2010; Xi et al., 2012). In this study, the tree topologies inferred from ML and BI using a restricted cp gene matrix were consistent with the trees inferred from the whole cp genome data (**Supplementary Figure S2**), but the support values for some nodes in the phylogenetic trees were lower. In this study, we proved that complete chloroplast DNA sequences were more effective than common protein coding genes for the phylogenetic reconstruction of Fagales, as evaluated by higher bootstrap values and posterior probabilities. Therefore, we suggest that complete chloroplast genomes should be used more regularly for inferring the backbone relationships among other ordinal clades of angiosperms, as well as for resolving the phylogenetic position of various questionable lineages.

## Conclusion

The complete chloroplast genome sequence of *M. rubra*, was determined using Illumina next-generation DNA sequencing technology. This is the first chloroplast genome sequenced in the Myricaceae family. The chloroplast genome of *M. rubra* shows a very similar size and organization comparing with the other sequenced angiosperms. The chloroplast genomes of Fagales species have experienced evolution at the gene level, rather than the genome level, because no significant structural changes are detected among their genomes. In addition, the examined genomes differ in size, and the detected genome size variations are mainly due to the length of intergenic spacers, instead of gene losses, gene pseudogenization, IR expansion or contraction. Inferred phylogenetic relationships based on the compete genome sequences from representatives of Fagales strongly support the placement of Myricaceae as sister to Juglandaceae. Furthermore, seven variable regions (*trn*H-*psb*A, *psb*A-*trn*K, *rps*2-*rpo*C2, *ycf*4-*cem*A, *pet*D-*rpo*A, *ndh*E-*ndh*G, and *ndh*A intron) and variable cpSSR loci identified among multiple individuals of *M. rubra* will be useful in future studies characterizing the population genetics of this species and investigating the domestication origin of Chinese bayberry.

## Author Contributions

LL, PL, CF, and XL conceived the ideas; LL and JW contributed to the sampling; LL performed the experiment; LL and RL analyzed the data. The manuscript was written by LL, PL, and KC.

## Conflict of Interest Statement

The authors declare that the research was conducted in the absence of any commercial or financial relationships that could be construed as a potential conflict of interest.

## Footnotes

1 http://www.phylo.org/

## Supplementary Material

FIGURE S1 The distribution and presence of simple sequence repeats (SSRs) in the cp genome of *M.rubra*-FJZS **(A)** and *M. rubra*-YNML **(B)**.

FIGURE S2 Phylogenetic tree reconstruction of 12 taxa of Fagales using maximum likelihood (ML) based on the 69 shared protein-coding genes sequences.
