# Supplementary figures and images for: The Complete Chloroplast Genome of Chinese Bayberry (Morella rubra, Myricaceae): Implications for Understanding the Evolution of Fagales
Source: Front Plant Sci. 2017 Jun 30;8:968. doi: 10.3389/fpls.2017.00968 (PMC5492642; doi:10.3389/fpls.2017.00968)

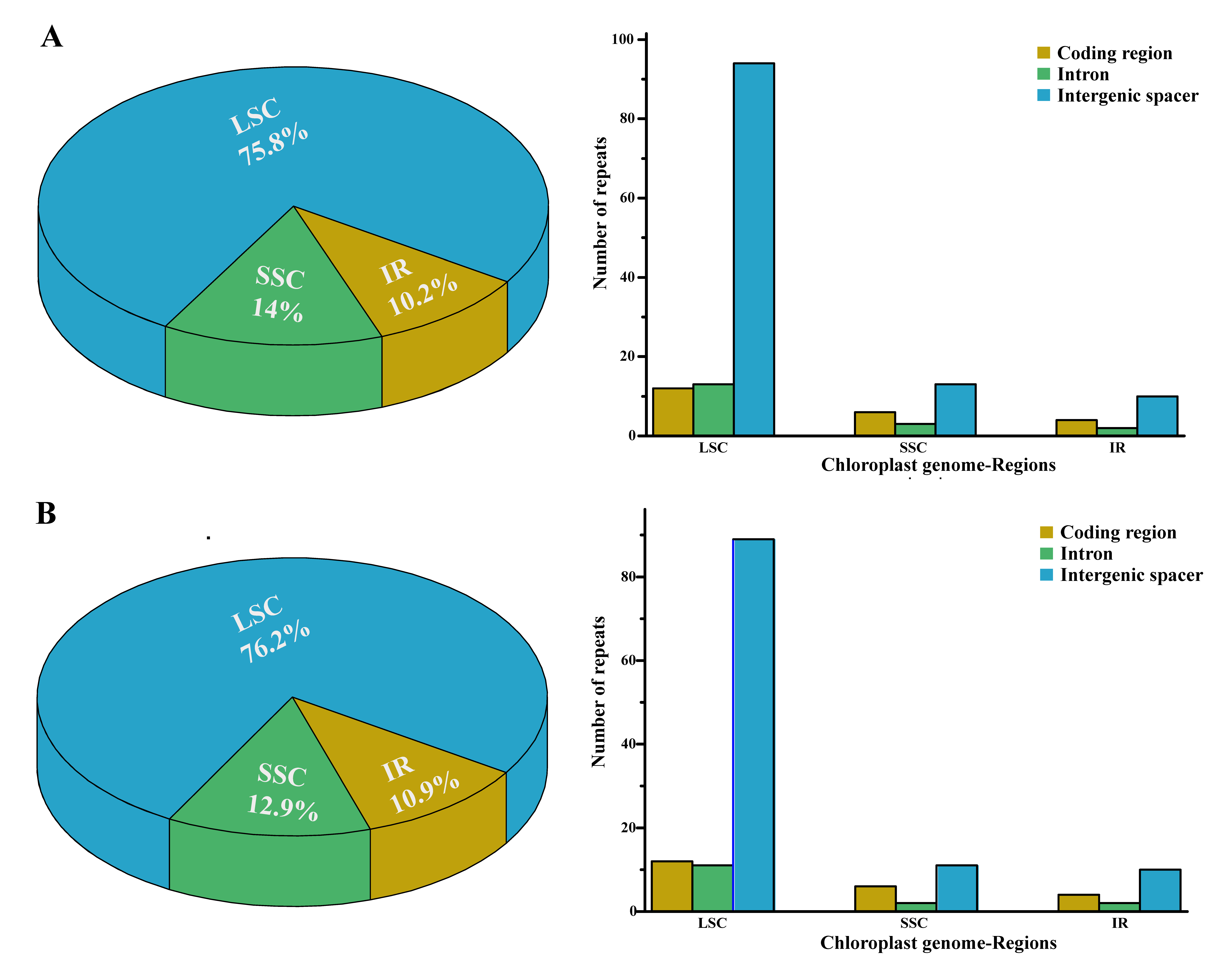

Supplement: FIGURE S1 — The distribution and presence of simple sequence repeats (SSRs) in the cp genome of M.rubra-FJZS (A) and M. rubra-YNML (B). [file Image_1.TIFF]
