# Supplementary material for: The Complete Chloroplast Genome of Chinese Bayberry (Morella rubra, Myricaceae): Implications for Understanding the Evolution of Fagales
Source: Front Plant Sci. 2017 Jun 30;8:968. doi: 10.3389/fpls.2017.00968 (PMC5492642; doi:10.3389/fpls.2017.00968)
Supplement: Supplementary file 3 [file Table_1.DOCX]

**Table S1| Accession numbers of chloroplast genomes used for phylogenetic analyses**

| Family | Genus | Taxon | Genbank No. | Source |
| --- | --- | --- | --- | --- |
| Myricaceae | *Morella* | *Morella rubra* (GZMZ) | KY476637 | This article |
| Myricaceae | *Morella* | *Morella rubra* (FJZS) | KY476636 | This article |
| Myricaceae | *Morella* | *Morella rubra* (YNML) | KY476635 | This article |
| Juglandaceae | *Juglans* | *Juglans regia* | KT870116 | Peng et al., 2016 |
| Betulaceae | *Ostrya* | *Ostrya rehderiana* | NC_028349 | Unpublished |
| Fagaceae | *Castanea* | *Castanea mollissima* | HQ336406 | Jansen et al., 2010 |
| Fagaceae | *Castanopsis* | *Castanopsis echinocarpa* | NC_023801 | Unpublished |
| Fagaceae | *Lithocarpus* | *Lithocarpus balansae* | NC_026577 | Unpublished |
| Fagaceae | *Quecus* | *Quecus rubra* | NC_020152 | Alexander and Woeste, 2014 |
| Fagaceae | *Trigonobalanus* | *Trigonobalanus doichangensis* | NC_023959 | Unpublished |
| Corynocarpaceae | *Corynocarpus* | *Corynocarpus laevigata* | HQ207704 | Atherton et al., 2010 |
| Cucurbitaceae | *Cucumis* | *Cucumis sativus* | DQ865976 | Chung et al., 2006 |
