# Supplementary material for: The Complete Chloroplast Genome of Chinese Bayberry (Morella rubra, Myricaceae): Implications for Understanding the Evolution of Fagales
Source: Front Plant Sci. 2017 Jun 30;8:968. doi: 10.3389/fpls.2017.00968 (PMC5492642; doi:10.3389/fpls.2017.00968)
Supplement: Supplementary file 4 [file Table_2.DOCX]

**Table S2| Repeated sequences in the Morella rubra-FJZS chloroplast genomes**

| Repeat no. | Repeat size (bp) | Repeat start 1 | Repeat start 2 | Type | Location of repeat 1 | Location of repeat 2 |
| --- | --- | --- | --- | --- | --- | --- |
| 1 | 30 | 136278 | 136309 | F | *rrn*5S/*rrn*4.5S* | *rrn*5S/*rrn*4.5S* |
| 2 | 30 | 134063 | 134063 | P | *ycf*1 | *ycf*1 |
| 3 | 30 | 125966 | 125966 | P | *ndh*A | *ndh*A |
| 4 | 30 | 114284 | 114284 | P | *ycf*1 | *ycf*1 |
| 5 | 30 | 114284 | 134063 | F | *ycf*1 | *ycf*1 |
| 6 | 30 | 112069 | 136309 | P | *rrn*4.5S/*rrn*5S* | *rrn*5S/*rrn*4.5S* |
| 7 | 30 | 112038 | 112069 | F | *rrn*4.5S/*rrn*5S* | *rrn*4.5S/*rrn*5S* |
| 8 | 30 | 112038 | 136278 | P | *rrn*4.5S/*rrn*5S* | *rrn*5S/*rrn*4.5S* |
| 9 | 30 | 47375 | 103109 | F | *ycf*3 | *rps*7/*trn*V-GAC* |
| 10 | 30 | 47375 | 145238 | P | *ycf*3 | *trn*V-GAC/rps7 |
| 11 | 30 | 42292 | 44516 | F | *psa*B | *psa*A |
| 12 | 30 | 39493 | 39509 | F | *psb*Z/*trn*G-GCC* | *psb*Z/*trn*G-GCC* |
| 13 | 30 | 38665 | 49159 | P | *psb*C/*trn*S-UGA* | *trn*S-GGA |
| 14 | 30 | 34576 | 34576 | P | *trn*T-GGU/*psb*D* | *trn*T-GGU/*psb*D* |
| 15 | 30 | 9335 | 49159 | P | *psb*I/*trn*S-GCU* | *trn*S-GGA |
| 16 | 31 | 126544 | 126544 | P | *ndh*A | *ndh*A |
| 17 | 31 | 117268 | 117268 | P | *ndh*F/*rpl*32* | *ndh*F*/rpl*32* |
| 18 | 31 | 35101 | 118295 | R | *trn*T-GGU/*psb*D* | *rpl*32/*trn*L-UAG* |
| 19 | 31 | 35101 | 118304 | C | *trn*T-GGU/*psb*D* | *rpl*32/*trn*L-UAG* |
| 20 | 31 | 24437 | 24438 | F | *rpo*C1 | *rpo*C1 |
| 21 | 31 | 11199 | 11222 | F | *trn*G-UCC/*trn*R-UCU* | *trn*G-UCC/*trn*R-UCU* |
| 22 | 31 | 9331 | 38661 | F | *psb*I/*trn*S-GCU* | *psb*C/*trn*S-UGA* |
| 23 | 32 | 154686 | 154707 | F | *ycf*2 | *ycf*2 |
| 24 | 32 | 132893 | 132893 | P | *ycf*1 | *ycf*1 |
| 25 | 32 | 93659 | 154707 | P | *ycf*2 | *ycf*2 |
| 26 | 32 | 93638 | 93659 | F | *ycf*2 | *ycf*2 |
| 27 | 32 | 93638 | 154686 | P | *ycf*2 | *ycf*2 |
| 28 | 32 | 6552 | 6620 | F | *rps*16/*trn*Q-UUG* | *rps*16/*trn*Q-UUG* |
| 29 | 33 | 125742 | 125742 | P | *ndh*A | *ndh*A |
| 30 | 34 | 124305 | 124305 | P | ndhG/ndhI* | *ndh*G/*ndh*I* |
| 31 | 37 | 47363 | 125504 | F | *ycf*3 | *ndh*A |
| 32 | 39 | 125502 | 145243 | P | *ndh*A | *trn*V-GAC/*rps*7* |
| 33 | 39 | 103095 | 125502 | F | *rps*7/*trn*V-GAC* | *ndh*A |
| 34 | 39 | 47363 | 103097 | F | *ycf*3 | rps7/trnV-GAC* |
| 35 | 39 | 47363 | 145241 | P | *ycf*3 | trnV-GAC/rps7* |
| 36 | 41 | 152260 | 152278 | F | *ycf*2 | *ycf*2 |
| 37 | 41 | 96076 | 152278 | P | *ycf*2 | *ycf*2 |
| 38 | 41 | 96058 | 96076 | F | *ycf*2 | *ycf*2 |
| 39 | 41 | 96058 | 152260 | P | *ycf*2 | *ycf*2 |
| 40 | 44 | 78780 | 78780 | P | *psb*I/*psb*N* | *psb*I/*psb*N* |
| 41 | 46 | 118292 | 118292 | P | *rpl*32/*trn*L-UAG* | *rpl*32/*trn*L-UAG* |
| 42 | 57 | 7002 | 7002 | P | rps16/*trn*Q-UUG* | *rps*16/*trn*Q-UUG* |
| 43 | 66 | 6552 | 6586 | F | *rps*16/*trn*Q-UUG* | *rps*16/*trn*Q-UUG* |
