# Supplementary material for: The Complete Chloroplast Genome of Chinese Bayberry (Morella rubra, Myricaceae): Implications for Understanding the Evolution of Fagales
Source: Front Plant Sci. 2017 Jun 30;8:968. doi: 10.3389/fpls.2017.00968 (PMC5492642; doi:10.3389/fpls.2017.00968)
Supplement: Supplementary file 5 [file Table_3.DOCX]

**Table S3 | Repeated sequences in the Morella rubra-YNML chloroplast genomes**

| Repeat no. | Repeat size (bp) | Repeat start 1 | Repeat start 2 | Type | Location of repeat 1 | Location of repeat 2 |
| --- | --- | --- | --- | --- | --- | --- |
| 1 | 30 | 136243 | 136274 | F | *rrn*5S/*rrn*4.5S* | *rrn*5S/*rrn*4.5S* |
| 2 | 30 | 134027 | 134027 | P | *ycf*1 | *ycf*1 |
| 3 | 30 | 125923 | 125926 | F | *ndh*A | *ndh*A |
| 4 | 30 | 117191 | 125926 | R | *ndh*F/*rpl*32* | *ndh*A |
| 5 | 30 | 114301 | 114301 | P | *ycf*1 | *ycf*1 |
| 6 | 30 | 114301 | 134027 | F | *ycf*1 | *ycf*1 |
| 7 | 30 | 112085 | 136274 | P | *rrn*4.5S/*rrn*5S* | *rrn*5S/*rrn*4.5S* |
| 8 | 30 | 112054 | 112085 | F | *rrn*4.5S/*rrn*5S* | *rrn*4.5S/*rrn*5S* |
| 9 | 30 | 112054 | 136243 | P | *rrn*4.5S/*rrn*5S* | *rrn*5S/*rrn*4.5S* |
| 10 | 30 | 75538 | 75541 | R | *clp*P | *clp*P |
| 11 | 30 | 47231 | 103130 | F | *ycf*3 | *rps*7/*trn*V-GAC* |
| 12 | 30 | 47231 | 145198 | P | *ycf*3 | *trn*V-GAC/*rps*7* |
| 13 | 30 | 42161 | 44385 | F | *psa*B | *psa*A |
| 14 | 30 | 38562 | 49014 | P | *psb*C/*trn*S-UGA* | *trn*S-GGA |
| 15 | 30 | 34482 | 34482 | P | *trn*T-GGU/*psb*D* | *trn*T-GGU/*psb*D* |
| 16 | 30 | 33703 | 33731 | F | *trn*E-UUC/*trnT*-GGU* | *trn*E-UUC/*trn*T-GGU* |
| 17 | 30 | 33051 | 46006 | C | *trn*D-GUC/*trn*Y-GUA* | *psa*A/*ycf*3* |
| 18 | 30 | 9246 | 49014 | P | *psb*I/*trn*S-GCU* | *trn*S-GGA |
| 19 | 31 | 126506 | 126506 | P | *ndh*A | *ndh*A |
| 20 | 31 | 125919 | 125921 | F | *ndh*A | *ndh*A |
| 21 | 31 | 117289 | 117289 | P | *ndh*F/*rpl*32* | *ndh*F/*rpl*32* |
| 22 | 31 | 117188 | 125925 | R | *ndh*F/*rpl*32* | *ndh*A |
| 23 | 31 | 88022 | 88043 | F | *rpl*22 | *rpl*22 |
| 24 | 31 | 9242 | 38558 | F | *psb*I/*trn*S-GCU* | *psb*C/*trn*S-UGA* |
| 25 | 32 | 154704 | 154725 | F | *ycf*2 | *ycf*2 |
| 26 | 32 | 132857 | 132857 | P | *ycf*1 | *ycf*1 |
| 27 | 32 | 125923 | 125923 | R | *ndh*A | *ndh*A |
| 28 | 32 | 117185 | 117188 | F | *ndh*F/*rpl*32* | *ndh*F/*rpl*32* |
| 29 | 32 | 93622 | 154725 | P | *ycf*2 | *ycf*2 |
| 30 | 32 | 93601 | 93622 | F | *ycf*2 | *ycf*2 |
| 31 | 32 | 93601 | 154704 | P | *ycf*2 | *ycf*2 |
| 32 | 32 | 34563 | 39477 | F | *trn*T-GGU/*psb*D* | *psb*Z/*trn*G-GCC* |
| 33 | 33 | 125798 | 125798 | P | *ndh*A | *ndh*A |
| 34 | 35 | 72602 | 72602 | P | *rps*18/*rpl*20* | *rps*18/*rpl*20* |
| 35 | 37 | 47219 | 125560 | F | *ycf*3 | *ndh*A |
| 36 | 39 | 125558 | 145203 | P | *ndh*A | t*rn*V-GAC/*rps*7* |
| 37 | 39 | 103116 | 125558 | F | *rps*7/*trn*V-GAC* | *ndh*A |
| 38 | 39 | 47219 | 103118 | F | *ycf*3 | *rps*7/*trn*V-GAC* |
| 39 | 39 | 47219 | 145201 | P | *ycf*3 | *trn*V-GAC/rps7* |
| 40 | 41 | 152278 | 152296 | F | *ycf*2 | *ycf*2 |
| 41 | 41 | 96039 | 152296 | P | *ycf*2 | *ycf*2 |
| 42 | 41 | 96021 | 96039 | F | *ycf*2 | *ycf*2 |
| 43 | 41 | 96021 | 152278 | P | *ycf*2 | *ycf*2 |
| 44 | 44 | 78571 | 78571 | P | *psb*T/*psb*N* | *psb*T/*psb*N* |
| 45 | 57 | 6948 | 6948 | P | *rps*16/*trn*Q-UUG* | *rps*16/*trn*Q-UUG* |
| 46 | 58 | 146859 | 146917 | F | *rps*7/*ndh*B* | *rps*7/*ndh*B* |
| 47 | 58 | 101441 | 146917 | P | *ndh*B/*rps*7* | *rps*7/*ndh*B* |
| 48 | 58 | 101383 | 101441 | F | *ndh*B | *ndh*B/*rps*7* |
| 49 | 58 | 101383 | 146859 | P | *ndh*B | *rps*7/*ndh*B* |
